# Peer support to improve diabetes care: an implementation evaluation of the Australasian Peers for Progress Diabetes Program

**DOI:** 10.1186/s12889-018-5148-8

**Published:** 2018-02-17

**Authors:** Zahra Aziz, Michaela A. Riddell, Pilvikki Absetz, Margaret Brand, Brian Oldenburg, On behalf of the Australasian Peers for Progress Diabetes Project Investigators, Brian Oldenburg, James A. Dunbar, Michaela A. Riddell, Prasuna Reddy, Virginia Hagger, Greg Johnson, Maximilian de Courten, Rory Wolfe, Rob Carter, Pilvikki Absetz, Anuar Zaini

**Affiliations:** 1 Melbourne School of Population & Global Health, the University of Melbourne, Melbourne, Australia; 2 Department of Epidemiology and Preventive Medicine, Monash University, Melbourne, Australia; 3 Department of Medicine, School of Clinical Sciences at Monash Health, Monash University, Melbourne, Australia; 4 School of Health Sciences, University of Tampere, FI-33014 Tampere, Finland; 5 Collaborative Care Systems Finland, Helsinki, Finland

**Keywords:** Type 2 diabetes, Self-management, Peer support, Implementation evaluation, RE-AIM framework, PIPE impact metric, Public health impact, Participant-level factors, Provider-level factors

## Abstract

**Background:**

Several studies have now demonstrated the benefits of peer support in promoting diabetes control. The aim of this study is to evaluate the implementation of a cluster randomised controlled trial of a group-based, peer support program to improve diabetes self-management and thereby, diabetes control in people with Type 2 Diabetes in Victoria, Australia.

**Methods:**

The intervention program was designed to address four key peer support functions i.e. 1) assistance in daily management, 2) social and emotional support, 3) regular linkage to clinical care, and 4) ongoing and sustained support to assist with the lifelong needs of diabetes self-care management. The intervention participants attended monthly group meetings facilitated by a trained peer leader for 12 months. Data was collected on the intervention’s reach, participation, implementation fidelity, groups’ effectiveness and participants’ perceived support and satisfaction with the intervention. The RE-AIM and PIPE frameworks were used to guide this evaluation.

**Results:**

The trial reached a high proportion (79%) of its target population through mailed invitations.

Out of a total of 441 eligible individuals, 273 (61.9%) were willing to participate. The intervention fidelity was high (92.7%). The proportion of successful participants who demonstrated a reduction in 5 years cardiovascular disease risk score was 65.1 and 44.8% in the intervention and control arm respectively. Ninety-four percent (94%) of the intervention participants stated that the program helped them manage their diabetes on a day to day basis. Overall, attending monthly group meetings provided ‘a lot of support’ to 57% and ‘moderate’ support to 34% of the participants.

**Conclusion:**

Peer support programs are feasible, acceptable and can be used to supplement treatment for patients motivated to improve behaviours related to diabetes. However, program planners need to focus on the participation component in designing future programs. The use of two evaluation frameworks allowed a comprehensive evaluation of the trial from the provider-, participant- and public health perspective. The learnings gained from this evaluation will guide and improve future implementation by improving program feasibility for adoption and acceptability among participants, and will ultimately increase the likelihood of program effectiveness for the participants.

**Trial registration:**

Australian New Zealand Clinical Trials Registry (ANZCTR) ACTRN12609000469213. Registered 16 June 2009.

## Background

The management and control of Type 2 Diabetes Mellitus (T2DM) is one of the major challenges confronting health systems and economies around the world [1]. As one of the predominant causes of premature death, loss of productivity and disability, diabetes was ranked among the top 15 most common causes of global disability-adjusted life years (DALYs) in 2013 [2]. The expected increase in diabetes cases by 55% (642 million people) by 2040, from the estimated 415 million people currently living with diabetes worldwide [3] will demand more health care resources placing further stress on health care systems. Hence, there is an urgent need to find new effective ways to improve diabetes self-management and control outcomes for people with diabetes (PWD) and reduce the public health impact of T2DM.

Furthermore, due to the complex nature of diabetes and its self-management, PWD require ongoing emotional, behavioural and social support to adopt and maintain self-care behaviours that help with the management of diabetes, reduce risk of complications and improve the quality of life [4]. However, many PWD find it difficult to receive such support from their families and friends [5]. Hence, a major opportunity exists to improve the management of T2DM through programs aimed at providing social and emotional support to PWD.

In recent years, peer support programs are seen as a promising approach in assisting PWD on an ongoing basis while emphasising sustained behaviour change required for improved self-management of chronic diseases [6]. Peer support refers to the provision of emotional and informational support, and practical assistance from people who have experiential knowledge of a condition [7–10], often in a way that can be mutually beneficial to those who have the same condition [11].

There is a large body of evidence supporting the positive impact of peer support on improving self-management in patients with T2DM such as adopting healthy behaviours due to increased knowledge and feeling of social connectedness [12–25]. Indeed, currently available evidence suggests that peer support can not only improve education and knowledge it can also lead to better health outcomes [4]. Furthermore, the “peer support” approach has been acknowledged by the World Health Organization (WHO) as a feasible, cost-effective and flexible intervention for improving diabetes care and outcomes [26]. However, less emphasis has been given to understanding program implementation.

Recognizing the need to build the evidence base for peer support, the American Academy of Family Physicians Foundation, initiated Peers for Progress (PfP) (http://peersforprogress.org) [27] in 2006. In 2009 PfP awarded 14 grants (including eight evaluation trials) in 9 countries with the objective to evaluate, demonstrate and promote peer support for diabetes around the world [11]. The PfP programme aimed to improve the evidence base for providing peer support to people with T2DM to improve self-management of their diabetes. The provision of community-based peer-led support, directed towards four key peer support functions, PfP programmes aimed to promote adoption and maintenance of lifestyle and behaviour changes. These key functions were 1) assistance in daily management, 2) providing social and emotional support, 3) promoting and supporting regular linkage to clinical care and community resources, and 4) provision of ongoing and sustained support to assist with the lifelong needs of diabetes self-care management [11]. The Australasian Peers for Progress - Diabetes Project (PfP-DP) [9], one of the eight funded evaluation trials, aimed to implement and evaluate a peer-led, group program to provide support to people with T2DM in Victoria, Australia.

Implementation science is “the scientific study of methods to promote the uptake of research findings” [28]. In addition to evaluating interventions’ effectiveness, there is a considerable need for more research on factors that enhance implementation of programs and optimise outcomes in a real-world setting. Without understanding whether a program was implemented as planned, there remains an uncertainty for program planners on how best to plan, design and implement the various components to achieve desired outcomes in future programs [29–31]. Research on the feasibility of effective peer support programs in diabetes is sparse [14, 17, 18] and there is still much to learn about how best to implement effective peer support programs.

The primary outcome of the PfP-DP was the predicted 5 year cardiovascular disease risk using the United Kingdom Prospective Diabetes Study (UKPDS) Risk Equation at 12 months. Secondary outcomes included clinical measures, quality of life, measures of support, psychosocial functioning and lifestyle measures. This implementation evaluation paper complements our earlier publication on the clinical and behavioural outcomes of the PfP-DP that demonstrated a small reduction in 5 year UKPDS risk in both the usual care and the intervention arms and the mean values for biochemical and anthropometric outcomes were close to target at 12-months [32].

Although published research supports the benefits of peer support in controlling diabetes, less attention has been paid to the translation of such research to everyday practice. There is a need for more research on factors that enhance implementation of programs and optimise outcomes for patients. In this paper we evaluate the implementation including the program’s reach, penetration and participation; the intervention’s fidelity; group effectiveness; perceived support by the participants, participants’ satisfaction, and willingness to continue to use the strategies learnt through this intervention; and barriers to participation in the program. In conducting this implementation evaluation, we have used Glasgow’s RE-AIM (Reach, Effectiveness, Adoption, Implementation, and Maintenance) framework [33], and Pronk’s PIPE (Penetration, Implementation, Participation, and Effectiveness) framework [34]. The details on the utilisation of these frameworks are included in the Methods section.

The learnings gained from this evaluation will guide and improve future implementation of peer support programs by improving program feasibility and acceptability, and, thereby, increase the likelihood of positive and beneficial effects for the participants.

## Methods

### Study design, setting and recruitment

The detailed study protocol is published elsewhere [9]. Briefly, we conducted a cluster randomised controlled trial of a peer-led group-based support program for people with T2DM. Victorian residents aged between 25 and 75 years with T2DM enrolled on the National Diabetes Services Scheme (NDSS) database for more than 12 months, were invited by mail to seek further information from the researchers about the PfP-DP. Administered by Diabetes Australia, NDSS is an initiative of the Australian Government for providing diabetes-related information and support services to people living with diabetes.

A total of 24 support groups consisting of 273 participants (10–11 group members and 1–2 peer leaders with diabetes per group) from 24 study locations (Local Government Authorities (LGAs)) within the state of Victoria, in Australia, were selected. The inclusion criteria for selecting LGAs was having a population of > 10,000 and NDSS pharmacy and /or community health/diabetes health care facility with links to Diabetes Australia-VIC. The 24 support groups were randomly allocated to equal numbers of intervention (12) and usual care (12) groups in each health region. Information on recruitment and allocation has been provided elsewhere [9]. All participants provided anthropometric, clinical (blood testing, including HbA1c, Cholesterol and LDL:HDL ratio) and survey data at baseline (prior to random allocation), and at 6 and 12 months follow-up. All participants received diabetes self-management education prior to allocation. The individual-level primary outcome was the cardiovascular disease (CVD) risk using the UKPDS risk equation [32, 35].

Blood glucose levels (HbA1C) were formally checked at baseline, 6 months and 12 months. HbA1c was chosen because it reflects glucose control over the prior 3 month period and is a better measure of long term glucose control. We compared HbA1c at baseline to that measured at 12 months, since this allowed us to compare those participants who had completed the program. Results of HbA1c, Cholesterol and LDL/HDL ratio were made available to all participants in each study arm at baseline, 6 months and 12 months, along with optimal targets for these measures. These results were also discussed during the group meetings and strategies to improve clinical measures were usually the focus of goal setting activities.

Blood glucose monitoring was key information provided in the participant’s handbook (p58–61) and participants were encouraged to implement self-monitoring especially if they were taking injectable medication.

This study received ethics approval from the Monash University Human Research Ethics Committee [9].

### Intervention program

The PfP-DP intervention was based on the four key peer support functions: 1) assistance in daily health management; 2) social and emotional support; 3) promotion and support of regular linkage to clinical care and community resources; and 4) provision of ongoing support to assist with diabetes self-management [11]. The protocol and main outcomes of the intervention program have been published previously [9, 32]. In brief, the intervention delivered ongoing support to intervention participants facilitated by a trained peer leader for 12 months. Support was implemented, through monthly group meetings, opportunities for shared activities outside of the group meetings, educational resources including diabetes education booklets and DVDs, each aimed at assisting group members to improve daily self-management of their diabetes. The peer group format allowed for ongoing social and emotional support to be provided by both peer leaders and group members. During the meetings, group members shared their experiences of living with and self-managing their diabetes and received ongoing support and guidance from peer leaders and other group members in setting and achieving manageable goals aimed at improving diabetes control. Group members were also encouraged and supported to improve and consolidate their relationship with their clinical care provider.

The program provided training and ongoing support to peer leaders in facilitating the group meetings through a 2 ½ day training program at the beginning of the intervention. A peer leader manual, which contained training material and other resources, was distributed to all leaders during training. The research team conducted weekly teleconferences with peer leaders to provide emotional, informational and appraisal support for peer leaders throughout the intervention period. The peer leaders were encouraged to attend at least one teleconference every month. The research team was available to provide additional support via teleconference and emails throughout the program.

Peer leaders and participants had access to a number of educational resources including a hard copy of a bi-monthly newsletter ‘Support Share Learn Live’ that was sent to all intervention participants during the intervention period. Other resources included access to a password-protected website containing further information, resources and education websites; a diabetes education manual; a PfP-DP specific Diabetes Self-Management Education (DSME) DVD; healthy eating book; pedometers; and other informational resources. A participant workbook was also delivered to all participants.

### Evaluation design

Provider- and participant-level implementation outcomes were evaluated using the RE-AIM [33] and PIPE [34] frameworks. The RE-AIM framework [33] includes five dimensions i.e. Reach (R), Effectiveness (E), Adoption (A), Implementation (I), Maintenance (M). The product of the five components determines the potential public health impact of an intervention [36]. The PIPE Impact Metric has four evaluation components i.e. Penetration (P), Implementation (I), Participation (P), Effectiveness (E) [34]. Similar to RE-AIM framework, each of the PIPE Impact Metric elements can be expressed as a coefficient, and the product of all elements represents the overall public health impact of an intervention. Both RE-AIM and PIPE frameworks employ provider-level as well as participant-level measures. In the PIPE framework these user levels are separate (Penetration and Implementation for provider and Participation and Effectiveness for participant). In the RE-AIM framework, several dimensions (i.e., Reach, Adoption, Maintenance) include both user levels, which makes it difficult to identify exactly which program element would need to be addressed to improve the program. Furthermore, several reviews have shown that the information on the five RE-AIM dimensions are largely underreported, particularly the ‘adoption’ and ‘maintenance’ dimensions [37–43], and that especially the dimensions of reach and adoption can be challenging to report with a valid denominator [44]. For both frameworks, the multiplicative model (R x E x A x I x M / P x I x P x E) of assessing the potential public health impact of a given intervention implies that if a program has a zero value on any dimension, that its overall public health impact will be zero [45].

**Fig. 1:**
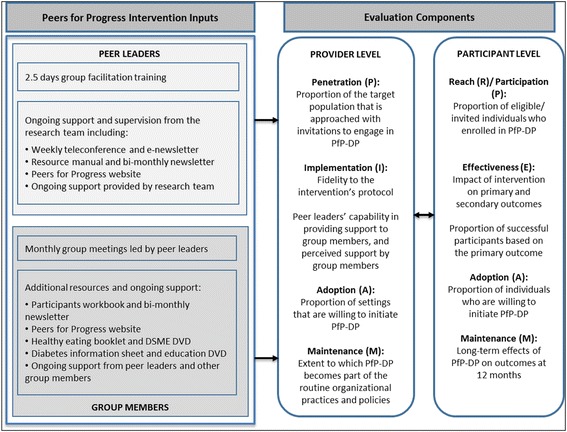
Logic model for PfP-DP evaluation design

Table 1 shows the definition of each of the evaluation components of RE-AIM and PIPE frameworks in the context of PfP-DP. Figure 1 describes how PfP-DP intervention’s program delivery inputs are evaluated as provider- and participant-level outcomes.

**Table 1:** Definition of RE-AIM and PIPE Components in the context of PfP-DP

RE-AIM [33]	PIPE [34]
*Reach (R)*	*Penetration (P)*
Proportion of eligible individuals who were willing to participate in PfP-DP.	Proportion of the target population that is reached with invitations to engage in PfP-DP.
*Effectiveness (E)*	*Implementation (I)*
The impact of PfP-DP on primary outcomes.	Fidelity with the intervention protocol.
	Participation by the potential program users in the delivery of intervention.
*Adoption (A)*	*Participation (P)*
Proportion of settings and individuals who were willing to initiate PfP-DP.	Proportion of invited individuals who enrolled in the program.
*Implementation (I)*	*Effectiveness (E)*
Fidelity with the intervention’s protocol.	Proportion of successful participants based on the primary outcome.
*Maintenance (M)*	
*Provider* – the extent to which PfP-DP became part of routine organisational practice.	
*Individual* - Long-term effects of PfP-DP on outcomes at 12 months.	
*Public Health Impact:* R x E x A x I x M	*Program Net Impact:* P x I x P x E

At the end of 12-months, all intervention participants were asked to complete a questionnaire designed to assess the implementation of PfP-DP in line with the four key peer support functions. The evaluation assessed participants’ interest and satisfaction with the intervention; group effectiveness; participants’ perceived support provided by peer leaders and group members; willingness to continue use of the strategies learnt through this intervention; and enablers of and barriers to adoption of the program.

## Results

This section follows the logic model for PfP-DP evaluation components as presented in Fig. 1. The results are presented for the provider- and participant-level measures using the evaluation components from the two frameworks.

### Provider-level measures

#### Penetration

The total number of NDSS registrants at the time of recruitment for selected LGAs was 102,492 including all types of diabetes, of which 9580 were deemed eligible, by the database custodians (i.e. agree to be contacted for research purposes), for invitations based on the above inclusion criteria. Letters of invitation were mailed to 7576 prospective participants with information about the Australasian PfP-DP and a detachable address form with reply-paid envelope to contact the project team if the individual was interested in receiving further information about participating in the study.

*Penetration Coefficient:* Penetration for this trial was calculated as 7576 / 9580 = **0.790 (79.0%)**.

#### Implementation

##### Intervention components delivered to peer leaders

Two and a half days of group facilitation training was delivered as planned. Of the 20 identified peer leaders for the intervention arm, 19 **(95%)** leaders completed the compulsory peer leader training aimed at preparing them for delivery of monthly group sessions to the intervention participants. The research team organised a weekly one-hour teleconference for 43 out of 47 weeks (**91%**). A total of 61% of peer leaders attended at least one teleconference per month which was the stated required minimum attendance for these teleconferences. The “Primus Inter Pares” *(English translation: “a first among equals; the senior or representative member of a group”)* weekly e-newsletter was developed by the research team and sent to peer leaders for 45 out of 47 weeks (96%) during the intervention period. The peer leaders had access to a range of educational resources during the intervention period.

The peer leaders were asked to rate how well the training program prepared them to provide peer support to their groups, on a scale of 1 to 10, where ten is ‘extremely well prepared’. Ninety-two percent (92%) of peer leaders rated 8 or more for the training, and 69% stated that the training prepared them “extremely well” in delivering peer support to their groups. All peer leaders indicated that the training manual and handouts were an ‘invaluable resource’ or ‘very helpful’ in delivering intervention and providing peer support to their respective groups.

Eighty-five (85%) leaders stated that the weekly teleconferences, weekly e-newsletters, and Peers for Progress Website provided them ‘moderate’ to ‘a lot of support’ in providing support to their group members. All peer leaders stated that they received ‘a lot of support’ from research staff in reference to the diabetes information, whereas, 75% stated that the research team provided a lot of administrative support. Seventy-seven percent (77%) of the peer leaders stated that they would like to continue to lead their peer support group after the completion of the formal intervention.

##### Intervention components delivered to group members by peer leaders in assistance with research team

Monthly group meetings were organised as scheduled for 11 of the planned 12 groups (**92%**) at a location convenient to the group members. For one group location allocated to receive the intervention, insufficient group members were recruited. This group was then combined with another location. These 11 groups met at least 12 times over the 12-month period. Nine out of 11 groups recorded an average attendance of 59% over the 12-month intervention. Two groups did not provide regular data on attendance.

*Implementation Coefficient:* We calculated the implementation coefficient by assessing the three major components delivered during the intervention i.e. Peer leaders training (**95%**), Weekly teleconference (**91%**), and Monthly group meetings (**92%**). Hence, the average proportion of the implementation components delivered during the intervention period according to the prescribed protocol was calculated as 92.7%. Hence, the implementation coefficient is determined to be **0.93 (93%)**.

#### Adoption

A total of 24 clusters (towns/cities) within 20 LGAs from 5 Regional and 3 Metropolitan health regions in the state of Victoria were recruited for the study. Twelve clusters were randomised and allocated to each control and intervention arms, out of which 11 clusters received the intervention.

*Adoption Coefficient:* Setting-level adoption was calculated as 11 / 12 = **0.916 (91.6%)**.

### Participant-level measures

#### Reach / participation

The detailed consort diagram and participants’ demographics have been published previously [32]. Briefly, out of 7576 mail invites, 294 were returned due to incorrect address details on the NDSS register. Of the remaining 7282 mails, expressions of interest were received by 501 (6.9%) individuals. Of these, 441 (88.0%) persons were eligible to enter the study and were sent recruitment packs including further information about the program and consent forms. Of these, 151 (34.2%) declined to participate, while 290 (65.7%) provided informed consent. Of 290, 17 (5.9%) withdrew their consent prior to random allocation of groups. Thus 273 individuals (94.1%) were available to participate in 24 groups. Of 273 individuals willing to participate, 33 volunteered and were deemed suitable for the role of peer leaders. Out of the remaining 240 participants, 120 (50%) were allocated into 12 groups in each of the intervention and control arm, governed by a random number generation process. Eighty-three percent and 82% of participants in the intervention arm completed 6-months and 12-months measurements respectively.

*Reach Coefficient:* Out of a total of 441 eligible individuals, 273 were willing to participate. Hence, the reach coefficient is calculated to be 273 / 441 i.e. **0.619 (61.9%)**.

*Participation Coefficient:* Out of a total of 7282 individuals who were sent mail invites to participate in the trial, 273 (3.7%) were enrolled. Hence, the participation coefficient is 273 / 7282 i.e. **0.037 (3.7%)**.

#### Effectiveness

The results on the effectiveness of the intervention with respect to the primary and secondary outcomes of the trial using the 5 year UKPDS risk score at baseline and 12 months have been published separately [32]. Briefly, the proportion of participants who showed improvement in the primary outcome i.e. reduction in 5 years UKPDS risk score was 0.651 (65.1%) in the intervention arm and 0.448 (44.8%) in the usual care group.

*Effectiveness Coefficient* = **0.651 (65.1%)**.

The findings of the effectiveness of the intervention delivery according to four key peer support functions are presented in Table 2. At the end of 12-months, two-thirds (*N* = 81, 67.5%) of the intervention participants completed the evaluation questionnaire.

**Table 2:** Effectiveness of the intervention delivery according to the four key peer support functions at 12 months

Key Functions (KF)	Participants’ evaluation
KF 1: Assistance in daily management of diabetes	• Ninety-four percent (94%) participants reported that the PfP-DP helped them manage their diabetes on a day to day basis ‘all the time’ (18%) and ‘to some extent’ (76%). Only 6% group members stated that the PfP-DP did not help them at all in managing their diabetes. • More than 90% of the group members stated that they were supported by their peer leaders in setting specific goals to improve their eating or exercise, in learning skills and behaviours to take care of their diabetes, and in solving problems that came up in taking care of their diabetes.
KF 2: Provision of promotion and social and emotional support	• Seventy-eight (78%) and 72% group members indicated that their peer leaders and other group members respectively supported them in dealing with stress. • However, about one-third (28%) felt that they could not call upon their peer leaders or other group members when they were feeling low or needed help from them. • Sixty-eight percent (68%) of participants when asked if they had received any ongoing social and emotional support in relation to their diabetes, besides participating in this program, responded that they had received additional support, mainly from their GPs and family members.
KF 3: Assistance in creating linkage with clinical care services	• Group members reported that the peer leaders reminded them to see their health care providers, very often (40%) to some of the time (38%), even when they were not sick. • Group members reported that peer leaders made referrals to community resources to help participants with clinical care from ‘very often’ (31%) to ‘some of the time’ (39%). • Seventy-eight percent (78%) of participants stated that their peer leaders asked them about problems with their medicines or their effects. • A total of 68% and 59% participants felt that their peer leaders and other group members respectively have helped them build better communication skills to use during their health care visits.
KF 4: Provision of ongoing support to assist with diabetes self-management	• Seventy-five percent (75%) and 63% of participants stated that they were able to contact and reach their peer leaders and other group members respectively, outside the monthly group meetings. • Seventy-five percent (75%) of the participants felt that their peer leaders maintained contact with them, and worked with them over time to help them manage their diabetes. • Ninety-five percent (95%) of the participants stated that peer leaders and other group members were able to contact them outside of the monthly group meetings. • The participants were asked about the extent to which the additional resources provided support. Diabetes Information Sheets, Healthy Eating Booklet, Diabetes Education and Manual, and Support Share Learn Live Newsletter provided a lot of support to 59, 55, 48 and 41% of participants respectively. • Overall, attending monthly group meetings provided ‘a lot of support’ to 57% and ‘moderate’ support to 34% of the participants. Only 8% of the group members felt that monthly group meetings provided only a little support in managing their diabetes.

According to RE-AIM, participant-level ‘adoption’ refers to reach and participation which is reported above. Similarly, participant-level ‘maintenance’ refers to long-term effectiveness, which is already reported under effectiveness. Hence, we do not report these two components separately.

#### Barriers to participation in program

At the end of 12-months, peer leaders were asked about the barriers that may have limited group members’ participation in group meetings. Five leaders reported barriers such as health issues and lack of time. Only one peer leader stated that the timings of the group meetings were not suitable for some participants. Similarly, group members were asked whether there were any factors that may have limited their participation in group meetings. More than half (56%) of the participants reported no barriers. Forty-four percent of respondents reported one or more factors including health issues (37%), lack of time/work commitments (37%), location/timings (37%), and issues in family/family commitments (14%).

#### Willingness to continue Behavioural change

At the end of 12 months, participants were asked whether they intended to use any strategies learnt in this program to self-manage their diabetes in the future. The majority of participants stated that they intended to continue to use strategies learnt in this program including healthy diet (91%), seeking timely clinical care (82%), regular exercise (77%), blood glucose monitoring (76%), and utilising social and emotional support (74%).

#### Public health impact / program net impact

As stated above, for both frameworks, the multiplicative model (R x E x A x I x M / P x I x P x E) of assessing the potential public health impact or program net impact of a given intervention implies that if a program has a zero value on any dimension, that its overall public health impact will be zero. Since we do not have coefficients of all five dimensions of RE-AIM, we use coefficients from four elements of the PIPE framework, to calculate the net program impact as the product of all four PIPE Impact Metric elements, i.e.

Penetration (0.790) X Implementation (0.927) X Participation (0.037) X Effectiveness (0.651) = 0.0176 **(1.76%)**.

Despite the very high rate of penetration (79%) and implementation (92.7%), and moderate effectiveness (65%), the net program impact (1.76%) of the PfP-DP intervention remains very low, a likely reflection of the low participation rate (3.7%).

## Discussion

In recent years, an increasing emphasis has been placed on the translation and implementation of research findings into real-world settings. This has resulted in an increase in the reporting of implementation evaluations in addition to reporting the primary and secondary outcomes. While the last 10 years has seen a big increase in the number of publications reporting the outcomes of peer support programs to improve diabetes outcomes [7, 12, 14–25], few have focussed on the ways in which such programs are affected by the local context and environment. Therefore, little is known about the implementation approaches of peer support programs. This paper reports the evaluation of the implementation of a peer-led, group-based program designed to provide support to people with T2DM in Victoria, Australia.

In undertaking this implementation evaluation, we have used Glasgow’s RE-AIM [33] framework that has been extensively used as an evaluation framework to guide both the design and evaluation of health program implementation [40]. We also use Pronk’s PIPE [34] Impact Metric that is also highly relevant to implementation. In using these two frameworks, we present a formal assessment of the net impact of health improvement programs conducted in real-world settings, with a clear distinction between the program providers’ and participants’ perspectives [46].

While assessing provider-level measures, we have reported trial penetration that reflects the efforts to reach each individual in the target population. As a community-based trial, we attempted to utilise common methods of recruitment which would be amenable to scale up such as invitation by mail, posters in various locations including clinics and pharmacies, and on Diabetes Australia -VIC website. Whilst achieving a very high rate of penetration (79%), reaching a large proportion of the target audience by mail invitations, the approach was low-intensity which may have limited influence in motivating the target population to participate. as compared to a well-designed social marketing campaign as observed in some other studies [47, 48]. Furthermore, postal invitation is a low-cost economic option for reaching target populations, the limitations of mail invitations are well documented [49]. We have conducted a systematic review of 38 real-world diabetes prevention programs from the last 15 years, using the PIPE Impact Metric framework and found that ‘high’ penetration into the target population with invitations to engage prospective participants in the program do not necessarily result in ‘high’ participation [46].

Interestingly, our calculation of reach (61.9%) and participation (3.7%) shows an enormous difference between these two concepts. According to the RE-AIM framework reach is calculated as the proportion of eligible individuals who were willing to participate (61.9%), whereas, the PIPE’s calculation of participation applies to the proportion of the invited individuals who enrolled in the program (3.7%). Although the later proportion might seem very low, it should be noted that one of the aims of our efficacy trial was to assess the feasibility of a community-based peer-led program in a real-world setting. Our approach was confined to letters to the target audience, inviting them to enrol in the study after satisfying eligibility and screening criteria. The low participation observed in our study is consistent with some diabetes prevention studies that have used mail invitations to recruit the potential participants [50, 51]. Furthermore, in our study, recruitment of peer leaders required their willingness to self-nominate for peer leadership and was tied to invitations to participate. In our experience, in other international studies, recruitment of peer leaders in India has been relatively straight forward [52, 53]. In Finland where we have initiated ongoing peer support for participants completing the nurse-facilitated GOAL lifestyle change groups to prevent T2DM [54], the peer-led support groups are built on the basis of an already existing platform, and the peer leaders have been slowly introduced to the role, along with strengthening of their self-efficacy. Furthermore, a recruitment strategy was designed in collaboration with the peer leaders and the nurse facilitators. This included a regular visiting schedule by the peer leaders to the GOAL groups to advertise the peer support meetings and invite new participants [54]. People living with T2DM may feel unable or ill-equipped to self-manage, and, to help others requires a higher level of confidence in self-management capacity. Future programs may benefit from distinct and detached strategies for participant recruitment and peer leader recruitment, and potentially higher intensity contact with the peer leaders before the program starts. Furthermore, several diabetes prevention studies have shown high participation rates where recruitment was carried out by referrals from physicians, general practitioners, or nurses from the participating health facilities [55, 56].. Future program planners should review the design and marketing strategies of the program.

In layman’s terms, ‘participation’ is considered as the willingness of participants to commence and adhere to the program. However, in our evaluation using PIPE metric, the participation by the potential users in the delivery of intervention falls under ‘implementation’. Hence, while calculating the implementation score, in addition to implementation fidelity, we specifically considered the participation in three major intervention delivery components i.e. “peer-leaders training”, “weekly teleconference”, and “monthly group meetings”. The individual implementation score varied from 91.5% for a weekly teleconference to 95% for the peer leader training. Program implementers succeeded in implementing the peer leader training, monthly group meetings and weekly teleconference as planned. The monthly group meetings provided support to the majority of participants in managing their diabetes. The participants’ perception of support received by peer leaders and other group members in reference to the four key peer support functions was also encouraging. A large majority of group members felt that they received assistance in the daily management of diabetes, which is an important finding. However, these findings should be viewed with caution as our results on the satisfaction of the program are based on the responses received by two-thirds of the participants only. The remaining one-third of the participants did not respond to the 12-month evaluation questionnaire. We also noted that 44% of these respondents reported factors that may have contributed to their lack of participation in the program, such as health issues, lack of time, work commitments, the location of monthly group meetings, timings of sessions, personal issues and family commitments. Future program implementation should endeavour to consider these barriers in the design and development of second generation peer support programs.

Our current calculation of PIPE Impact Metric shows a small overall impact of 1.76%, which, although low, should be placed in the context of what may be a ‘theoretically’ realistic impact in the real-world settings. The PfP-DP has been moderately effective with an individual success rate of 65% in the intervention arm, but the effectiveness of the overall program is dramatically diminished due to the very low participation discussed above. While our recruitment approach via the national register for PWD in Australia was feasible and cost effective, such an approach may not be suitable for reaching those individuals with poor diabetes control and those experiencing other complications, who would likely benefit the most from such a program. In a hypothetical scenario for a similar program, keeping all the PIPE coefficients the same as PfP-DP except imputing a participation rate of 20% instead of 3.7%, as in our program, the overall program impact could increase to almost 10%, while similar increase in effectiveness would only give a 2% impact. Hence, the incremental benefit from increased effectiveness of intervention is bound to be much smaller than the incremental benefit from effective recruitment.

The assessment of the overall public health impact of an intervention could be invaluable in guiding and informing future translation and dissemination of the results into policy and practice. However, the measurement of public health impact of real-world interventions is a complex issue and is not reported widely. Compernolle and colleagues [37] conducted a systematic review of 35 interventions which used the RE-AIM framework to assess the potential public health impact of evidence-based multi-level interventions to improve obesity-related behaviours in adults. The authors reported that only three studies out of 35 reported on all five dimensions of RE-AIM. Authors then calculated the average RE-AIM score to assess the potential public health impact of interventions and concluded that one of the three interventions had achieved the highest potential public health impact, despite its low participation rate. This is in contrast to our calculation of program impact using the PIPE Impact metric, where the PfP-DP had a very low net impact due to the low participation rates. However, it should be noted that Compernolle et al. [37] calculated the public health impact by taking the average of all RE-AIM components, whereas, we have calculated the net impact by taking the product of the four dimension of PIPE (as per the definition of PIPE Impact Metric) [34] and hence achieved 1.76%. If we had used the same method, the public health impact of the PfP-DP would have been 60%. Hence, in our view, Compernolle et al. presents an overestimation of the overall public health impact.

While the RE-AIM framework has continued to evolve and has been increasingly used to facilitate the translation of research findings, there are some limitations in applying reporting criteria for all five dimensions of RE-AIM [39]. In our view, the RE-AIM definition of individual-level maintenance is equivalent to long-term effectiveness. Traditionally many research trials do not collect follow-up data at approximately 12 months after intervention cessation, and this may not be practical in a research context. Similarly, like other authors [44] we found the definition of reach and individual-level adoption overlapping and we could not differentiate between the two. In our view, ‘adoption’ should relate to the willingness to adopt the strategies taught in the intervention (e.g., goal setting, improving diet, increasing physical activity, etc.)

In a recent systematic review of diabetes prevention programs, we analysed 38 studies, choosing the PIPE Impact Metric for evaluation rather than the RE-AIM. We determined the PIPE framework to be more appropriate, informative and less complex in evaluating the degree of program impact on its objectives. This framework considers both provider related factors i.e. penetration and implementation, and participant related factors i.e. participation, and effectiveness [46]. More examples of program evaluation using the PIPE metric is needed so that similar programs could be compared based on the overall PIPE metric. Mapping the components from the two models into provider- and participant-level factors for this evaluation, allowed us to undertake a comprehensive evaluation of PfP-DP from providers’ as well as participants’ perspective.

When combined with outcome evaluation, implementation evaluation can contribute to an evidence base for wider implementation and scale up of research programs, potentially achieving population-level impact; and for facilitating the translation of research trials into successful public health programs in real-world settings. This is also true for trials that report only modest effects as achieved in our study. Our findings show that a group-based, peer support program to assist people in improving diabetes control, is feasible and acceptable as measured by participants. Participant satisfaction, perceived support, usefulness of program training/strategies and resources, willingness to use strategies learnt in the program in future as well as the moderate primary outcome measured by success rate support feasability and acceptability of our programme. Furthermore, the participation rate although adequate for research purposes was low in terms of public health impact. If disseminated, future programs should focus on better participation strategies such as better communication strategies, clearly stated benefits of the program, and outreach strategies of sufficient intensity to ensure maximum participation. The design and development stages of the Australasian PfP-DP did not invite participants into program planning. Future programs aimed at self-management of T2DM may need to more effectively engage with target groups. Without a participatory approach, we might not be able to overcome issues related to low participation rates for such programs. Further refinements are needed for improving the participation rates in future programs. More research is needed to bring structured and effective peer support programs to PWD to assist them in improving self-management and reducing subsequent morbidity and mortality.

## Conclusion

The Australasian PfP–DP was implemented in accordance with the four key peer support functions i.e. 1) assistance in daily health management; 2) social and emotional support; 3) promotion and support of regular linkage to clinical care and community resources; and 4) provision of ongoing support to assist with diabetes self-management. The use of two evaluation frameworks allowed a comprehensive evaluation of the PfP-DP from the provider-, participant- and public health perspective. Our findings suggest that peer support programs can be feasible and acceptable and that they have the potential to create a positive impact on the quality of life of people with T2DM, which ultimately increases the likelihood of program effectiveness for the participants. However, program planners should enhance focus on the participation component in designing future programs. More examples of program evaluation using PIPE metric are needed so that similar programs could be compared based on the overall PIPE metric. The results of this evaluation will help in understanding peer-support programs better and will elucidate improvements for future replications of such programs both in Australia and in other similar settings.

## Abbreviations

CVD: Cardio vascular disease
DALYs: Disability-adjusted life years
DSME: Diabetes self-management education
LGAs: Local Government Authorities
NDSS: National diabetes services scheme
PfP-DP: Australasian peers for progress - diabetes project
PIPE: Penetration, implementation, participation, effectiveness
PWD: People with diabetes
REAIM: Reach, effectiveness, adoption, implementation, maintenance
T2DM: Type 2 diabetes mellitus
UKPDS: United Kingdom prospective diabetes study
WHO: World Health Organization
